# A life threatening problem in infants: supratentorial epidural hematoma

**Published:** 2009-04-25

**Authors:** AV Ciurea, A Tascu, FM Brehar, L Nuteanu, R Rizea

**Affiliations:** ‘Bagdasar Arseni’ Clinical Emergency Hospital, BucharestRomania

**Keywords:** Epidural hematoma, Infant, Pallor, Children Coma Scale(CGS), Traumatic Infant Neurological Score(TINS), Outcome

## Abstract

Traumatic epidural hematoma (EDH) represents a rare head injury complication in infants.Its diagnosis can be quite challenging because its clinical presentation is usually subtle and nonspecific.Authors present a study on 30 infants with epidural hematoma (EDH) admitted in the Pediatric Department of Neurosurgery of the ‘Bagdasar–Arseni’ Clinical Hospital in the period of 1990–2007 (17 years).The most common symptom was irritability, which occurred in 16 cases (53.3%), of our patients. Pallor in all cases (100%) and subgaleal hematoma in 20/30 (66.6%) of the patients. These were the most common clinical signs that occurred upon admission; both of them represent signs of significant clinical importance. Surgical evacuation via craniotomy was required in 26/30 (86.6%) of our patients, while 4/30 (13.3%) of the patients were managed conservatively. The mortality rate was 6.6% in our series, whilst the long–term morbidity rate was 3.3%.

EDH in infants represents a life–threatening complication of head injury, which requires early identification and prompt surgical or conservative management depending on the patient's clinical condition, the size of EDH, and the presence of a midline structure shift on the head's CT scan.

## Introduction

Traumatic epidural hematoma (EDH) constitutes a rare clinical and pathological entity in children. It has been estimated that EDH represents 2–3% of all head injuries in the pediatric population, and the incidence of EDH is even less encountered among infants under the age of 12 months [1–5]. However, the specific characteristics of this group of patients and the subtle presenting symptomatology of EDH makes it difficult to be diagnosed and often challenging to be managed. Furthermore, the criteria for using surgical evacuation vs. conservative management have remained ill defined. Thus, the lack of any guidelines regarding the appropriate management of EDH in pediatric patients and particularly in infants makes the management of this specific group of patients even more complicated. The reported mortality rates associated with EDH in infants and children are different from one clinical series to another [6–10], and this broad variation is an indicative for the regrettable absence of a widely accepted protocol for the management of these patients. In our current communication, we are presenting our data from a series of infants diagnosed with traumatic EDH and managed in our institutions; emphasis has been put on their presenting symptoms and signs, their diagnostic significance, and on the long–term outcome of these patients.

## Materials and methods

Thirty cases (14 girls and 16 boys) with supratentorial EDH aged between 0𠄴1 years old have been admitted in the hospital in the last 17 years. The Institutional Review Board of our institutions approved the study, and the data analysis was performed in accordance to the Health Insurance Portability and Accountability Act regulations. The hospital, outpatient clinical charts, and radiographic studies of all patients were meticulously reviewed. Patients with spontaneous EDH or patients with EDH of unknown etiology, as well as patients with infratentorial EDH, were excluded from our current study.

The mean age of the series was 10 months. The etiology was: fall from other level 18 cases (60.0%), domestic accidents 6 cases (20.0%), car accidents 4 cases (13.3%), and child abuse 2 cases (6.6%). The Children Coma Scale (CCS) at admission ranged between 13–15 in 11 cases (36.6%), 9𠄴12 in 13 cases (43.3%) and 4–8 in 6 cases (20.0%) (Table 1).

**Table 1:** CCS admitting scores in our series

**Children Coma Scale (CCS)**	**Number of patients**	**Percent**
13–15	11	36.6%
9–12	13	43.3%
4–8	6	20.0%

The clinical status was characterized by pallor in all cases (100%), irritability 16 cases (53.3%), somnolence 13 cases (43.3%), fullness of fontanel 12 cases (40.0%), hemiparesis 8 cases (26.6%), seizures 5 cases (16.6%), third nerve paresis 4 cases (13.3%), fever 3 cases (10.0%), hemorrhagic shock 3 cases (10.0%). The comatose state was noticed in 6 cases (20.0%). Each infant's admitting laboratory workup included measurements of complete blood count, serum electrolytes, blood urea nitrogen, creatinine, serum glucose, and prothrombin and partial thromboplastin times. No associated coagulopathy was detected in this series. Long bone radiographic survey and ophthalmologic examination, including but not limited to fundoscopic examination, were obtained whenever suspicion of child abuse was raised.

All cases were investigated by CT scan within 3–6 hours from the traumatic event. Twenty cases (66.6%) presented associated subgaleal hematoma and 22 cases (73.3%) had cranial fracture. Patient management was either surgical or conservative based on the infant's clinical condition, Children Coma Scale (CCS) score, TINS score, evidence of midline shift on the initial head CT scan, and size of the EDH.

In 26/30 cases (86.6%) the size of the EDH was more than 2cm in midline shift. These underwent to immediate surgical intervention. Surgical management consisted of craniotomy under general endotracheal anesthesia and removal of the underlying hematoma.

The remaining 4/30 cases (13.386.6%) with low size EDH without mass–effect and CCS of 13–15 were managed conservatively. Conservative management consisted of close observation in either a neonatal or a pediatric intensive care environment, with heart rate, respiratory rate, and oxygen saturation monitoring. Moreover, frequent neurological clinical examinations and serial head CT scans (initially at admission, and then at 12, 24, 48, and 72 h unless neurological changes dictated otherwise) were done.

The follow-up time in our series ranged between 12–60 months (meaning 36.8 months). The patient's follow–up included clinical examination with detailed neurological examination, imaging studies (head CT in all patients), electrophysiological studies (EEG) in 10/30 (33.3%) patients, and neuropsychological evaluation in 6/30 (20.0%) patients. The Glasgow Outcome Scale (GOS) was used in order to evaluate the outcomes in our series.

## Results

The age of patients ranged between 1 day and 12 months, with a mean age of 10 months. There were 14 girls and 16 boys. Concerning the lateralization of the hematoma in our study, 18/30 (60.0%) were located on the right side, and the remaining 40.0% were located on the left side, while the temporo-parietal area was the most common anatomical location of the hematoma (Table 2).

**Table 2:** Anatomic location of the EDH in our series

**Anatomic location of EDH**	**Number of patients**	**Percent**
Temporo–parietal	14	46.6%
Parietal	7	23.3%
Temporal	5	16.6%
Frontal	3	10.0%
Fronto–temporo–parietal	1	3.3%

In order to obtain a better assessment of the clinical status of infants at admission, we have also used the Trauma Infant Neurological Scores (TINS). It is interesting to note that both our patients who died had a TINS score of 10 upon their admission.

**Table 3:** Our data regarding TINS admitting scores

**TINS**	**Number of patients**	**Percent**
10	2	6.6%
9	1	3.3%
8	1	3.3%
6	3	10.0%
5	6	16.6%
1–4	18	60.0%

**Figure 1:**
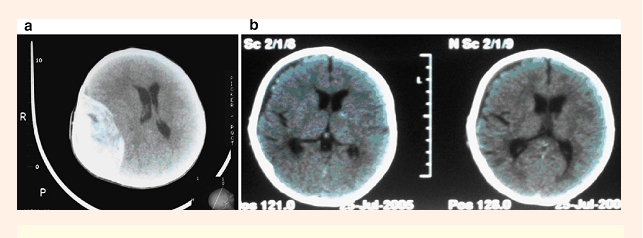
Preoperative(1a) and postoperative(1b) head CT scans in a 5–month–old infant with EDH

The CT scan performed at admittance showed that the size of the EDH was more than 2cm in 26/30 (86.6%) patients, between 1–2cm in 2/30 (6.6%), while in 2/30 (6.6%) the EDH was less than 1cm in its largest diameter. Emergent surgical evacuation (Figure 1) under general endotracheal anesthesia was performed in 26/30 cases (86.6%) of our patients, while the remaining 4/28 (13.3%) were conservatively treated. The hospitalization in our series ranged between 3 and 12 days (mean 4.2 days, median 5 days).

In 27/30 cases (90.0%) the evolution was good (GR); the neurological status was improved after surgical treatment and only medical treatment. In two cases (6.6%) the patients died postoperatively of acute anemia. The median period of the follow–up was 3 years. No significant changes were observed at 24 months after the treatment. No long–term posttraumatic sequels were encountered except for three cases (two were surgically treated and one conservatively) where the patients suffered from rare episodes of seizures. One of these patients (3.3%) has remained with long–term morbidity on anticonvulsant medications, and the other two are seizure–free with no medications. Neuropsychological evaluation data were available in six of our patients. Analysis of these data demonstrated normal psychomotor development in all these children.

## Discussion

It is well known that acute epidural hematomas in children, and especially in infants, represent a quite rare and potentially life–threatening complication resulting from head injuries [11–13]. Furthermore, epidural hematomas in infants constitute a different clinical entity than the ones in adults due to their nonspecific clinical presentation and the inability of infants to communicate. The most common mechanism of injury in infants, in our series, was domestic fall from height in 60.0% of our cases. Our finding is in agreement with previous reports stating that this is the prevailing cause of such injuries [11–15]. *Beni–Adani et al. *[14], in their infantile series, reported that in 63.6% of their cases, fall from height was responsible for the development of an acute epidural hematoma. Similarly, *Pasaoglu et al.* [12] found that fall was the most common underlying mechanism in 63% of their pediatric cases, and *Ersahin et al.* [13] identified fall as the most common mechanism of injury in 62% of their pediatric cases. On the contrary,* Rocchi et al.* found that traffic–related accidents were the most common cause of EDH in their series; this finding might be explained by the fact that they reported on children and not solely on infants [11]. It has been demonstrated since before that falls represent the most common cause of EDH in infants and children up to 5 years old [15]. It has also been emphasized that even minor head injuries can lead to the development of an acute EDH in infants [14 –16]; this is despite of the fact that falls from more than 1m height carry worse prognoses [14].

The appearance of moderate pallor in all our patients upon admission was a characteristic sign of major diagnostic significance. Similarly, *Pasaoglu et al.* [12] reported that pallor and anemia occurred in 90% of their infantile cases. Anemia, associated with pallor, has been identified since before as an important laboratory finding in infants with acute EDH [2–7–13 –17–18]. Cephalhematoma was another common clinical sign, which occurred in 66.6% of our cases. Likewise, *Beni- Adani et al.* reported the existence of cephalhematoma in the vast majority of their patients [14]. It is interesting to note that we did not observe lucid intervals in any of our patients. Pasaoglu et al. [12], however, reported that 32% of their pediatric patients presented with a typical lucid interval. *Ersahin et al.* [13] reported that 24% of their patients who presented with a lucid interval died, while their mortality rate among patients presenting without a lucid interval was significantly lower.

Past studies demonstrate that the presence of a lucid interval can easily mislead or delay the accurate diagnosis and the prompt management of an underlying EDH [14–19]. Various clinical examination-grading systems have been proposed for the evaluation of infants [11–14 –20]. *Ersahin et al.* [13], and *Pasaoglu et al.* [12] concluded in their studies that the Glasgow Coma Scale (GCS) scoring system accurately assessed neurological condition even in infants and was associated with outcome in a statistically significant fashion. Similarly,* Rocchi et al.*[11] found that the preoperative neurological status examined either by GCS or CCS had a significant impact on outcome. In their study, *Beni-Adani et al.* stated that the widely used CCS system included parameters which were difficult to interpret and score, and they therefore, suggested a new scoring system (TINS) that included more objective parameters [14]. We used their system in our study because we share their concerns regarding the applicability of the GCS and the CCS grading system for evaluating infants. We found high TINS scores (10) in both of our patients who eventually died. Unfortunately, the limited number of our cases makes the extraction of any statistically meaningful conclusions regarding the outcome predictive value of TINS scoring scale impossible.

The temporo–parietal area was the most common anatomical location of EDH in our study. Our findings demonstrated that anatomical location was not associated with outcome. Similarly, *Ersahin et al.* [13], and *Pasaoglu et al.* [12] reported that temporo–parietal and temporal regions were the most common locations in their series; again, no relationship between the anatomical location of EDH and the outcome was established. Previous investigators, however, have postulated that temporal location might contribute to increased mortality due to the predisposition to uncal herniation [21–24].

The treatment of choice in the majority of acute traumatic EDHs remains to be surgical evacuation via a flap craniotomy [11–14]. 86.6% of the cases in our series were surgically evacuated, while o nly 13.4% were treated conservatively. However, it must be emphasized that our centers are tertiary neurosurgical centers, so our population might represent a preselected one, consisting of patients referred to our facilities due to their larger sized hematomas or poor clinical condition. The criteria for selecting patients for conservative vs. surgical treatment have remained controversial [25 –28]. *Chen et al.* suggested that a hematoma volume larger than 30mL, with thickness of more than 15mm, and a midline shift more than 5mm constitute strong indications for surgical evacuation [25].

Our mortality rate (6.6%) is similar to that reported by *Beni-Adani et al.* [14] (9.1%) in their infantile series. *Rocchi et al.* [11] reported a mortality rate of 5.5% in pediatric patients having solely epidural hematomas. Similarly, *Ersahin et al.* [13] reported a mortality of 6% in their pediatric series. *Pasaoglu et al.* [12], in their series, reported a mortality rate of 12% in patients with pure epidural hematomas. However, a large number of their patients were treated in the pre–CT era, a fact that could significantly delay the diagnosis and the prompt management of EDH [12]. Our long–term morbidity rate was only 3.3% (one patient with chronic seizures, well controlled with medications). The limited data of our study regarding the neuropsychological development of these infants revealed no long–term consequences. Nevertheless, the psychomotor and cognitive development of infants sustaining EDH is an area that requires further study.

## Conclusion

Authors consider that EDH in infants is an emergency and can be managed by surgery in cases with poor neurological status or in cases in which the neuroimagistic signs show brain compression, a brain shift of more than 0,5 cm or the size of the hematoma bigger than 2 cm. In these cases, the surgical treatment must be performed early in order to obtain a good outcome. The CT scan must be performed as soon as possible after admission, usually in the first 3 hours to realize the optimal management in EDH in infants. The patients' neurological condition, the size of the EDH, and the presence of midline shift on head CT scans are the most commonly employed criteria for making a decision between surgical or conservative treatment.
